# Editorial: Genomics and metagenomics approaches for food value chain quality, safety, and product development

**DOI:** 10.3389/fmicb.2022.1056796

**Published:** 2022-11-10

**Authors:** Caroline Barretto, Jerome Combrisson, Nicholas Bokulich

**Affiliations:** ^1^Nestlé Research Center, Lausanne, Switzerland; ^2^Mars, McLean, VA, United States; ^3^Institute of Food, Nutrition, and Health, ETH Zürich, Zurich, Switzerland

**Keywords:** genomics, metagenomics, food safety, food quality, product development

This Research Topic collects different contributions highlighting how genomics and metagenomics techniques are revolutionizing investigations of microbial activity along the complete food value chain, from farm to fork, advancing insight into functional roles of microbial strains and consortia in food quality and safety. Advances in 2nd- and 3rd-generation DNA sequencing technologies and computational methods for microbial data science have paved the way for multi-omics characterization of microbial communities in agriculture and food production, with a myriad of applications including pathogen tracing, functional characterization, and microbial ecology. Genomics applied to food production will become a new trend for routine food chain management, but critical assessment and future direction are needed, including better standardization, interoperability, and quality control in food applications.

Food safety and quality management regimes typically employ traditional methods for microbial detection and characterization, which have known limitations including levels of detection and resolution. Genomics and metagenomics methods have numerous benefits that overcome these limitations, offering potential applications that could improve food safety, quality, and sustainability. Nevertheless, other limitations must be addressed, including standardization and best practices for food applications.

The goal of this Research Topic is to critically evaluate applications of sequencing technologies and analysis techniques in food quality and safety throughout the food value chain. This encompasses genomics and metagenomics applications that span raw materials production to final products.

Manuscripts include applications of 2nd- and 3rd-generation sequencing technologies for genomics and metagenomics in food microbiology, e.g., genomics of foodborne pathogens or beneficial food microbes, bioinformatics techniques and digital resources with specific applications in food quality and safety, foods as model systems for microbial ecology, and genomics and metagenomics applications in food fermentation.

The first article of this Research Topic (Djemiel et al.) gives an overview of -omics technologies contribution to the monitoring of the soil microbial quality, notably exploring the use of various methods to investigate the microbial diversity of soils. While showing the general principles, methodologies, specificities and benefits, the article also describes some of the challenges that need to be overcome to achieve a better understanding of soil microbial complexity.

Raw material monitoring is key to avoiding pathogens entering production in food facilities. The risks of having *Cronobacter* or *Salmonella* pathogens entering the food chain is highlighted in the article from Parra-Flores et al. The authors show the benefit of using whole genome sequencing (WGS), sequence typing prediction, determination of serotypes, and antibiotic resistance profiles on those pathogens.

Borges et al. describes how omics techniques have revolutionized food microbiology including bio-preservation to improve the microbial safety of various foods, the future being food microbiome engineering by considering food as a complex and dynamic microbiome, opening the field of innovation of production.

The genomes of six *Weissella* species were sequenced and analyzed by Fanelli et al. allowing the authors to demonstrate the strong carbohydrate utilization capabilities of the sequenced strains. The increasing availability of the genomic sequences of the *Weissella* species contributes to improving the knowledge about this genus and identifying the features defining its role in fermentative processes.

Wambui et al. reports a comprehensive genomic analysis of the *Clostridium estertheticum* complex (CEC) belonging to a diverse group of bacteria associated with spoilage of chilled vacuum-packed meat. The comprehensive comparative genomics of 50 genomes supports the reconstruction and comparison of intra- and interspecies metabolic pathways linked to meat spoilage.

Braley et al. addressed the gut microbiota of meat-producing animals as being one of the most important sources of surface contamination of processed carcasses, a concern for both food safety and for the shelf life of pork meat products. Based on their observations, even with optimal primary processing practices, gut microbiota modifications may not have any profound effects on carcass microbiota. This is an important learning for the control of bacterial carcass surface contamination in slaughterhouses.

Using both culture-dependent methods and 16S rRNA gene sequencing, Liao and Wang illustrated that there was no significant difference in bacterial abundance, diversity, and composition of bacterial communities in three brands of Romain Lettuce (RL) on the “Use By” dates (UBD) and on the 5 days after the “Use By” dates (UBD5), suggesting that the microbial quality of RL remained the same at two storage time points. Interestingly, bacterial communities present in RL samples were influenced by “brand” and “harvesting season” factors.

Ishiya et al. determined the whole genome sequences of three bacterial strains, named FNDCR1, FNDCF1, and FNDCR2, isolated from a practical nata-de-coco producing bacterial culture. The 16S rDNA sequence and phylogenetic analysis revealed that all strains belonged to a different clade within the *Komagataeibacter* genus.

The aim of the Nouws et al. study was to assess the potential benefits of WGS compared with conventional molecular methods currently used in the investigation of Staphylococcal food poisoning (SFP) outbreaks. Complete Staphylococcal enterotoxin (*se*) gene profiling of isolates from various sources, and relatedness between isolates were investigated using WGS, enabling complete *se* gene profiling with high performance, in contrast to PCR-based *se* gene detection for which primers sometimes showed to be non-specific.

Finally, Buytaers et al. assessed new methodologies to investigate food samples in the event of food-borne outbreaks. The authors present a proof-of-concept using shiga toxin-producing *Escherichia coli* for a strain-level metagenomics food-borne outbreak investigation method using the MinION and Flongle flow cells from Oxford Nanopore Technologies. Strain-level characterization could be achieved, linking the food containing a pathogen to the related human isolate of the affected patient, by means of a SNP-based phylogeny, with the use of long reads applying a bioinformatics method.

Together, this Research Topic demonstrates applications of genomics and metagenomics approaches to better elucidate the role of microbial ecology on food safety and quality assurance from farm to fork. In addition to showcasing new technologies and applications for food chain monitoring, several articles in this Research Topic highlight the need for discussion within the international scientific community to overcome several challenges in utilizing and translating these technologies, including the need for well-validated international standards for harmonization and interoperability.

## Author contributions

CB, JC, and NB wrote the manuscript. All authors made a direct and intellectual contribution to the work and approved the final version for publication.

## Conflict of interest

Author CB is employed by Nestlé. Author JC is employed by Mars. The remaining author declares that the research was conducted in the absence of any commercial or financial relationships that could be construed as a potential conflict of interest.

## Publisher's note

All claims expressed in this article are solely those of the authors and do not necessarily represent those of their affiliated organizations, or those of the publisher, the editors and the reviewers. Any product that may be evaluated in this article, or claim that may be made by its manufacturer, is not guaranteed or endorsed by the publisher.

